# Comparative Effectiveness of Intravenous Tenecteplase versus Alteplase in Posterior Circulation Ischemic Stroke: A Systematic Review and Meta‐Analysis

**DOI:** 10.1002/brb3.71831

**Published:** 2026-10-08

**Authors:** Shaheer Bin Shafiq, Ammad Uddin, Muhammad Hussain, Ghulam Taha Khan, Muhammad Hassan, Muhammad Junaid Razzak, Mominah Majid, Faisal Islam, Umer Ahmed, Ahmed Anwaar Uddin, Hasibullah Aminpoor, Muhammad Ahmed

**Affiliations:** ^1^ Department of Medicine Dow University of Health Sciences Karachi Pakistan; ^2^ Dr Ishrat Ul Ibad Khan Institute of Dentistry, Ojha Campus Dow University of Health Sciences Karachi Sindh Pakistan; ^3^ Faculty of Medicine Kabul University of Medical Sciences “Abu Ali Ibn Sina” Kabul Afghanistan

**Keywords:** tenecteplase, alteplase, posterior circulation ischemic stroke, stroke, reperfusion

## Abstract

**Background:**

Posterior circulation ischemic stroke (PCIS) is associated with substantial morbidity and mortality, and the optimal intravenous thrombolytic strategy in this subgroup remains unclear.

**Methods:**

We searched databases for studies comparing intravenous tenecteplase with alteplase in adults with PCIS. The primary outcome was functional independence at 90 days (mRS 0–2). Secondary outcomes included other functional outcomes, reperfusion, hemorrhagic complications, and mortality.

**Results:**

Five studies involving 997 patients were included. Regarding efficacy outcomes, tenecteplase was associated with a significantly greater likelihood of achieving an ambulatory functional outcome at 90 days (mRS 0–3) [RR 1.29, 95% CI 1.03–1.60; *p =* 0.02] and improved pre‐EVT perfusion [RR 2.18, 95% CI 1.06–4.46; *p =* 0.03]. No significant differences were observed for excellent functional outcome (mRS 0–1) [RR 1.19, 95% CI 0.91–1.56; *p =* 0.20], functional independence (mRS 0–2) [RR 1.10, 95% CI 0.89–1.37; *p =* 0.39], or post‐EVT perfusion [RR 1.12, 95% CI 0.89–1.40; *p =* 0.34]. Regarding safety outcomes, no significant differences were observed between tenecteplase and alteplase for symptomatic intracranial hemorrhage (sICH) [RR 0.89, 95% CI 0.36–2.22; *p =* 0.81], any intracranial hemorrhage [RR 1.02, 95% CI 0.59–1.78; *p =* 0.94], parenchymal hematoma [RR 0.76, 95% CI 0.24–2.38; *p =* 0.64], or 90‐day mortality [RR 0.88, 95% CI 0.66–1.17; *p =* 0.39].

**Conclusion:**

Tenecteplase may improve reperfusion and ambulatory recovery without increasing hemorrhagic complications or mortality compared with alteplase in PCIS, although larger PCIS‐specific studies are needed.

## Graphical abstract

Visual summary of the comparative efficacy and safety of intravenous tenecteplase versus alteplase in posterior circulation ischemic stroke.

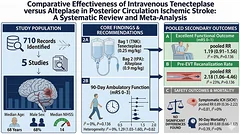

## 1 Introduction

Posterior circulation ischemic stroke (PCIS), which affects the vertebrobasilar arterial territory, accounts for approximately one‐fifth to one‐quarter of ischemic strokes and disproportionately involves the brainstem and cerebellum, with basilar artery occlusion (BAO) carrying particularly high morbidity and mortality (Schneider et al. 2023). PCIS presents distinct diagnostic and therapeutic challenges because its clinical manifestations are heterogeneous and frequently under‐recognized, while imaging and reperfusion paradigms developed largely for anterior circulation stroke may have limited applicability to vertebrobasilar pathology (Ng 2022, Go 2015). Although population‐level stroke mortality continues to be influenced by modifiable vascular risk factors such as hyperlipidemia (Ahmed et al. 2025), once PCIS occurs, the immediate clinical priority is timely recognition and effective reperfusion. This is particularly important because injury to vital brainstem structures and the unfavorable natural history of BAO can result in severe disability or death. Accordingly, optimizing acute reperfusion therapy specifically for posterior circulation stroke remains an important clinical priority.

Intravenous thrombolysis is an important component of acute reperfusion therapy in eligible patients with ischemic stroke. Alteplase has traditionally been used for this purpose, whereas tenecteplase, a genetically modified tissue plasminogen activator with greater fibrin specificity and a longer half‐life that permits single‐bolus administration, has emerged as an attractive alternative (Campbell et al. 2018). Recent randomized trials and large pragmatic studies have demonstrated comparable or non‐inferior functional outcomes, improved early reperfusion in selected populations, and generally similar rates of symptomatic intracranial hemorrhage (sICH) and mortality with tenecteplase compared with alteplase (Campbell et al. 2018; Wang et al. 2023; Wang et al. 2024; Muir et al. 2024; Kvistad et al. 2022; Alamowitch et al. 2023). However, most of these studies enrolled predominantly anterior circulation or mixed anterior and posterior circulation populations. Consequently, the extent to which these findings can be directly extrapolated to PCIS remains uncertain, and posterior circulation‐specific data on functional outcomes, hemorrhagic complications, mortality, and recanalization remain comparatively limited or inconsistently reported (Wang et al. 2023; Wang et al. 2024; Muir et al. 2024; Kvistad et al. 2022).

Current guideline recommendations and scientific statements acknowledge tenecteplase at 0.25 mg/kg as a reasonable alternative to alteplase in several acute ischemic stroke settings, particularly in patients with large‐vessel occlusion, while recognizing persistent uncertainty across specific patient subgroups (Alamowitch et al. 2023; Kim et al. 2025). Similarly, although an increasing number of meta‐analyses have compared tenecteplase with alteplase in acute ischemic stroke, most have evaluated broader ischemic stroke populations rather than focusing specifically on the posterior circulation or vertebrobasilar territory (Palaiodimou et al. 2024). Thus, an important evidence gap remains regarding the comparative efficacy and safety of these thrombolytic agents specifically in PCIS. Accordingly, we performed a systematic review and meta‐analysis of adults with neuroimaging‐confirmed PCIS to compare intravenous tenecteplase with standard‐dose intravenous alteplase. We aimed to clarify their comparative effects on functional recovery, reperfusion, hemorrhagic complications, and mortality and thereby provide a posterior circulation‐specific synthesis to inform clinical decision‐making.

## 2 Methods

This meta‐analysis was conducted in accordance with the Preferred Reporting Items for Systematic Reviews and Meta‐Analyses (PRISMA) 2020 guidelines (Page et al. 2021). The review protocol was prospectively registered in PROSPERO ID: (CRD420261340568) in order to ensure methodological transparency and rigor.

### 2.1 Search Strategy

Electronic databases, including PubMed, Embase, Scopus, and Cochrane Central Register of Controlled Trials, were systematically searched to identify relevant studies, covering the period from their inception to February 14, 2026. The search was designed to identify studies comparing the efficacy and safety of intravenous tenecteplase versus intravenous alteplase in adult patients with PCIS. Both Medical Subject Headings (MeSH) and relevant free‐text keywords were used, including “posterior circulation stroke”, “vertebrobasilar stroke,” “basilar artery occlusion,” “acute ischemic stroke,” “tenecteplase,” “TNK,” “alteplase,” “tPA,” “thrombolysis,” “functional independence,” “modified Rankin Scale,” “symptomatic intracranial hemorrhage,” “mortality,” and “recanalization.” Boolean operators were used to combine the search terms appropriately. The detailed search strategy is presented in Table S1. In addition, the reference lists of all included studies and of previously published systematic reviews and meta‐analyses on this topic were manually screened to identify any further eligible studies.

### 2.2 Study Selection

The articles identified through the comprehensive search were exported to Rayyan AI, and duplicates were removed. Titles and abstracts of the remaining studies were independently reviewed by two authors (M.H. and M.J.R.), and full‐text articles were retrieved for further assessment. Any discrepancies that arose were resolved by a third author (A.U.). There were no restrictions regarding publication date or language. Study eligibility was defined using the PICOS framework as follows. Population (P): adults aged 18 years or older with acute ischemic stroke involving the posterior circulation or vertebrobasilar territory confirmed by neuroimaging. Intervention (I): intravenous tenecteplase at any dose. Comparator (C): standard‐dose intravenous alteplase (0.9 mg/kg). Outcomes (O): at least one of the efficacy or safety outcomes prespecified in Section 2.4. Study design (S): randomized controlled trials, including post hoc and secondary posterior circulation analyses of randomized controlled trials, and comparative observational cohort studies. We excluded studies based on the following criteria: (Schneider et al. 2023) pediatric populations; (Ng 2022) non‐human studies; (Go 2015) case reports, reviews, editorials, letters, conference abstracts without sufficient extractable data, and non‐comparative studies; and (Ahmed et al. 2025) failure to report relevant posterior circulation subgroup data or any prespecified outcome.

### 2.3 Data Extraction and Quality Assessment

Data from included studies were extracted independently by the two authors (G.T.K. and A.U.). Author details, study characteristics, study design, country, sample size, patient demographics, baseline clinical characteristics, intervention details, comparator details, and outcome data were extracted onto a predesigned Excel spreadsheet. Any disagreement between the two authors was settled through consultation with the third independent author (S.B.S.). Quality assessment of the included studies was conducted independently by two authors (S.B.S. and M.M.). The Cochrane Risk of Bias tool for randomized trials (RoB 2) was applied to evaluate the methodological quality of randomized controlled trials (RoB 2: A revised Cochrane risk‐of‐bias tool for randomized trials 2026), while observational studies were assessed using the Newcastle–Ottawa Scale (NOS) (Newcastle‐Ottawa Scale (NOS) 2026).

### 2.4 Outcomes and Definition

The primary outcome was functional independence at 90 days, defined as a modified Rankin Scale (mRS) score of 0–2. Secondary efficacy outcomes included excellent functional outcome (mRS 0–1), ambulatory functional outcome (mRS 0–3), and successful pre‐EVT and post‐EVT perfusion based on study‐level definitions of recanalization or reperfusion. Safety outcomes included symptomatic intracranial hemorrhage (sICH), any intracranial hemorrhage, any parenchymal hematoma, and 90‐day mortality. All outcomes were analyzed as defined by the individual studies. An mRS shift analysis was not feasible because complete ordinal 90‐day mRS data for the posterior circulation population were unavailable; therefore, the reported dichotomized mRS thresholds were analyzed separately.

### 2.5 Statistical Analysis

All statistical analyses were conducted using Review Manager (RevMan) version 5.4 (The Nordic Cochrane Centre, Copenhagen). Dichotomous outcomes were pooled as relative risks (RRs) with 95% confidence intervals using the Mantel–Haenszel method under a random‐effects model with the DerSimonian–Laird estimator of between‐study variance (Ahmed et al. 2026). A random‐effects model was selected a priori because clinical and methodological heterogeneity was anticipated. Statistical heterogeneity was assessed using the I^2^ statistic, with values of approximately 25%, 50%, and 75% indicating low, moderate, and high heterogeneity, respectively (Higgins et al. 2003). Rare safety outcomes, including symptomatic intracranial hemorrhage, any intracranial hemorrhage, and parenchymal hematoma, were pooled using the Mantel–Haenszel random‐effects method. For studies with zero‐event cells, RevMan 5.4 applies continuity correction according to its default handling of sparse binary data. No studies were excluded because of zero‐event data. Publication bias was not formally assessed because fewer than ten studies contributed to each outcome; this limitation was acknowledged.

Subgroup analyses were performed according to study design and stroke population. For study design, studies were categorized as randomized controlled trials (RCT)/mixed‐PCIS studies versus observational studies. For the stroke population, studies were categorized as mixed‐PCIS versus basilar artery occlusion (BAO)‐only studies. Because these classifications resulted in an identical partition of the included studies, a single subgroup analysis was used to address both sources of heterogeneity. Subgroup differences were assessed using the χ^2^ test for interaction.

### 2.6 Certainty of Evidence

The certainty of evidence was assessed using the GRADE framework, and a summary of findings table was generated with GRADEpro software (Zhang et al. 2019). Certainty was evaluated across risk of bias, inconsistency, indirectness, imprecision, and publication bias, with outcomes rated as high, moderate, low, or very low certainty.

## 3 Results

A visual summary of the study design, key efficacy and safety outcomes, and overall clinical interpretation is provided in the Graphical Abstract.

### 3.1 Search Results

A total of 710 records were identified through systematic database searches across PubMed, EMBASE, Scopus, and Cochrane CENTRAL. After removing duplicates, 641 records were selected for screening based on the title and abstract. Of these, 634 studies were excluded based on title and abstract review, following which 7 potentially relevant articles were reviewed based on full text. Ultimately, 5 studies met our inclusion criteria (Alemseged et al. 2021; Bala et al. 2024; Cimflova et al. 2024; Karamchandani et al. 2025; Wolman et al. 2025). The study selection process is summarized in the PRISMA flow diagram, Figure 1.

**FIGURE 1:**
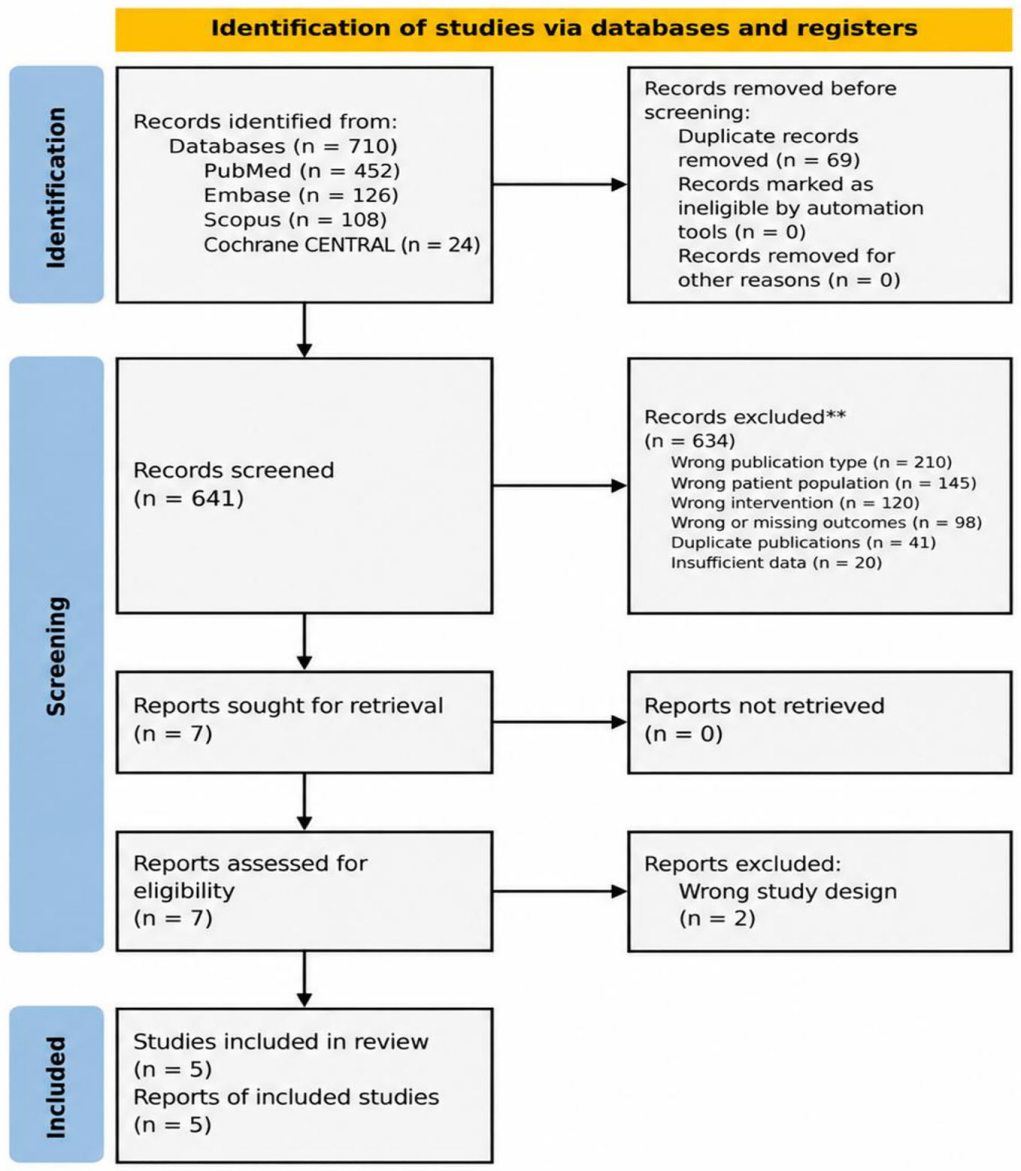
Prisma flowchart.

### 3.2 Study Characteristics

Five studies were included in the analysis, comprising a total of 997 adult patients, of whom 449 were assigned to the tenecteplase group and 548 received alteplase. The pooled mean age was approximately 70.5 years in the tenecteplase group and 69.8 years in the alteplase group. Male participants accounted for 243/449 (54.1%) in the tenecteplase group and 324/548 (59.1%) in the alteplase group. Baseline stroke severity, assessed using the National Institutes of Health Stroke Scale (NIHSS), was comparable between groups, with median scores generally ranging from 7 to 20 in the tenecteplase group and 7 to 22 in the alteplase group, reflecting a spectrum from moderate to severe stroke. Diabetes mellitus was reported in selected studies, with proportions ranging from 11% to 18% in the tenecteplase group and 29% to 36% in the alteplase group, indicating a relatively higher burden of diabetes among patients receiving alteplase. The remaining baseline characteristics are presented in Table 1.

**TABLE 1:** Baseline and study characteristics.

Characteristic	Alemseged (2021)		Bala (2024)		Cimflova (2024)		Karamchandani (2025)		Wolman (2025)	
	TNK	tPA	TNK	tPA	TNK	tPA	TNK	tPA	TNK	tPA
**Study descriptors**										
Population represented by baseline data	BAO cohort		Overall MeVO cohort^†^		Posterior circulation subgroup		BAO cohort		BAO EVT cohort	
Study design	Pooled retrospective analysis (RCTs + registry)		Secondary analysis of phase III pragmatic RCT		Post‐hoc analysis of phase III pragmatic RCT		Retrospective multicenter cohort		Retrospective multicenter cohort	
Country/setting	Multinational		Canada		Canada		United States		United States	
Thrombolytic dose, mg/kg	0.25 or 0.40	0.90	0.25	0.90	0.25	0.90	0.25	0.90	0.25	0.90
Total patients, n	19	91	236	219	59	77	75	88	60	73
Age	77 ± 14	67 ± 14	77 (65–84)	76 (66–84)	72 (65–82)	71 (60–81)	64 (50–72)	66 (57–79)	64 (50–72)	64 (55–78)
Male sex, n (%)	10 (53)	60 (66)	129 (54.7)	116 (53.0)	40 (67.8)	44 (57.1)	35 (46.7)	55 (62.5)	29 (48.3)	49 (67.1)
Baseline NIHSS	20 (5–32)	15 (7–32)	8 (5–14)	8.5 (6–14)	7 (4–16)	7 (4–12)	16 (9–28)	21 (11–26)	19 (11–28)	22 (13–28)
Endovascular thrombectomy, n (%)	19 (100)	91 (100)	47 (19.9)	40 (18.3)	12 (20.3)	16 (20.8)	75 (100)	88 (100)	60 (100)	73 (100)
Follow‐up	90 d	90 d	90–120 d	90–120 d	90 d	90 d	90 d	90 d	90 d	90 d
**Comorbidities / medical history**										
Hypertension, n (%)	11 (58)	53 (58)	NR	NR	NR	NR	48 (64.0)	70 (79.5)	38 (63.3)	58 (79.5)
Diabetes mellitus, n (%)	2 (11)	26 (29)	NR	NR	NR	NR	12 (16.0)	30 (34.1)	11 (18.3)	26 (35.6)
Dyslipidemia / hypercholesterolemia, n (%)	7 (37)	25 (27)	NR	NR	NR	NR	34 (45.3)	54 (61.4)	27 (45.0)	48 (65.8)
Atrial fibrillation, n (%)	5 (26)	23 (25)	NR	NR	NR	NR	18 (24.0)	17 (19.3)	14 (23.3)	14 (19.2)
Smoking history, n (%)	1 (5)	17 (19)	NR	NR	NR	NR	30 (40.0)	52 (59.1)	23 (38.3)	44 (60.3)
Coronary/cardiac disease, n (%)	4 (21)	20 (22)	NR	NR	NR	NR	13 (17.3)	19 (21.6)	10 (16.7)	16 (21.9)
Previous TIA or stroke, n (%)	NA	NA	NR	NR	NR	NR	11 (14.7)	11 (12.5)	9 (15.0)	10 (13.7)
Premorbid mRS ≤1, n (%)	NA	NA	NR	NR	NR	NR	66 (88.0)	78 (88.6)	54 (90.0)	65 (89.0)
**Physiology / workflow**										
Systolic blood pressure, mm Hg	NA	NA	NR	NR	NR	NR	150 (135–165)	153 (130–179)	151 (134–169)	150 (129–177)
Diastolic blood pressure, mm Hg	NA	NA	NR	NR	NR	NR	84 (74–91)	84 (73–95)	84 (72–94)	83 (73–94)
Glucose, mg/dL	NA	NA	NR	NR	NR	NR	133 (111–165)	137 (117–166)	130 (111–166)	137 (120–176)
Onset‐to‐needle time, min	181 (93–203)	160 (130–200)	136 (98–190)	130 (95–191)	136 (100–213)	141 (108–197)	NR	NR	124 (97–182)	138 (103–201)
Needle‐to‐puncture time, min	48 (40–71)	110 (51–185)	NR	NR	NR	NR	NR	NR	78 (45–136)	94 (56–161)
Onset‐to‐puncture time, min	NR	NR	NR	NR	269 (152–355)^*^	174 (142–328)^*^	NR	NR	NR	NR

*Note*: Values are reported from the source manuscript baseline/subgroup tables used for extraction. Male sex n (%) was calculated from treatment‐arm totals where the source table reported female sex only.

Abbreviations: TNK: tenecteplase; tPA: alteplase; BAO: basilar artery occlusion; MeVO: medium vessel occlusion; EVT: endovascular thrombectomy; NA: not applicable; NR: not reported.

^a^Bala (2024) baseline characteristics represent the overall MeVO cohort, which included anterior‐ and posterior‐circulation occlusions; they are not restricted to a posterior‐circulation subgroup.

^b^Data for patients undergoing EVT.

### 3.3 Quality Assessment

The risk of bias assessment demonstrated overall favorable methodological quality among the included studies. Among the three included randomized trials, all have a low overall risk of bias across the evaluated domains according to the ROB2 Tool Table S2. Notably, while data from *post hoc* studies were utilized for the pooled quantitative analyses, the risk of bias was assessed using their respective original, primary trials. For observational studies assessed using the NOS method, quality ranged from 7 to 9 stars, indicating moderate to high quality. Overall, the included evidence demonstrated adequate methodological rigor, with no major concerns identified in the quality assessment in Table S3.

### 3.4 Primary Outcomes

#### 3.4.1 MRS 0–2

Patients in both groups demonstrated comparable good functional outcomes, with no statistically significant difference observed (RR 1.10, 95% CI 0.89–1.37; *p =* 0.39; I^2^ = 40%) Figure 2.

**FIGURE 2:**
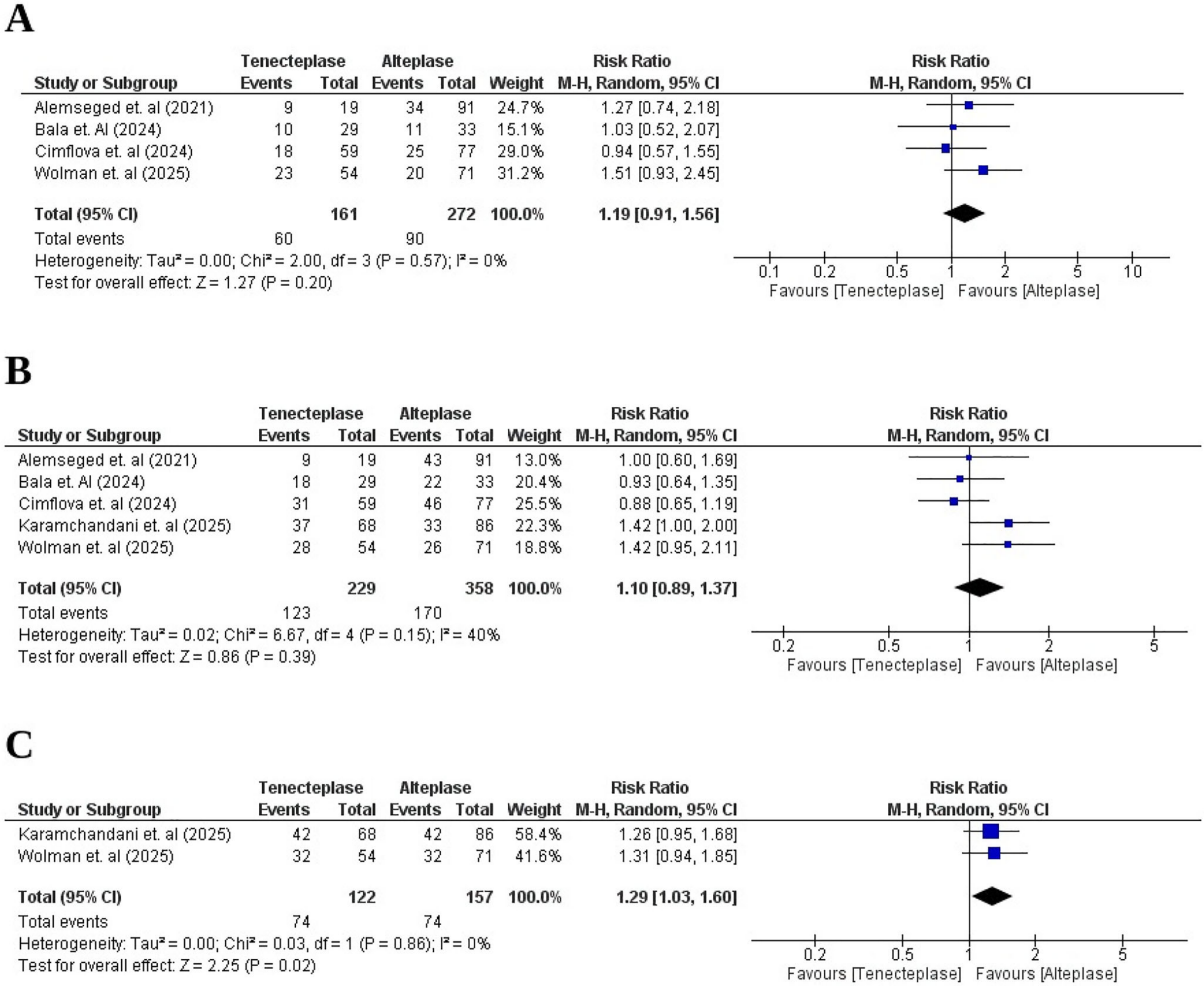
Forest plot comparing 90‐day functional outcomes between tenecteplase versus alteplase.

### 3.5 Secondary Outcomes

#### 3.5.1 MRS 0–1

Regarding excellent recovery, findings favored tenecteplase over alteplase, though the difference failed to reach statistical significance (RR 1.19, 95% CI 0.91–1.56; *p =* 0.20; I^2^ = 0%) Figure 2.

#### 3.5.2 (MRS 0–3)

A statistically significant difference was observed for ambulatory functional outcomes, favoring tenecteplase over alteplase (RR 1.29, 95% CI 1.03–1.60; *p =* 0.02; I^2^ = 0%) Figure 2. However, this analysis included two studies with a relatively small sample size.

#### 3.5.3 Pre‐EVT Perfusion

A statistically significant increase in successful pre‐EVT perfusion was observed in the tenecteplase group (RR 2.18, 95% CI 1.06–4.46; *p =* 0.03; I^2^ = 23% Figure 3.

**FIGURE 3:**
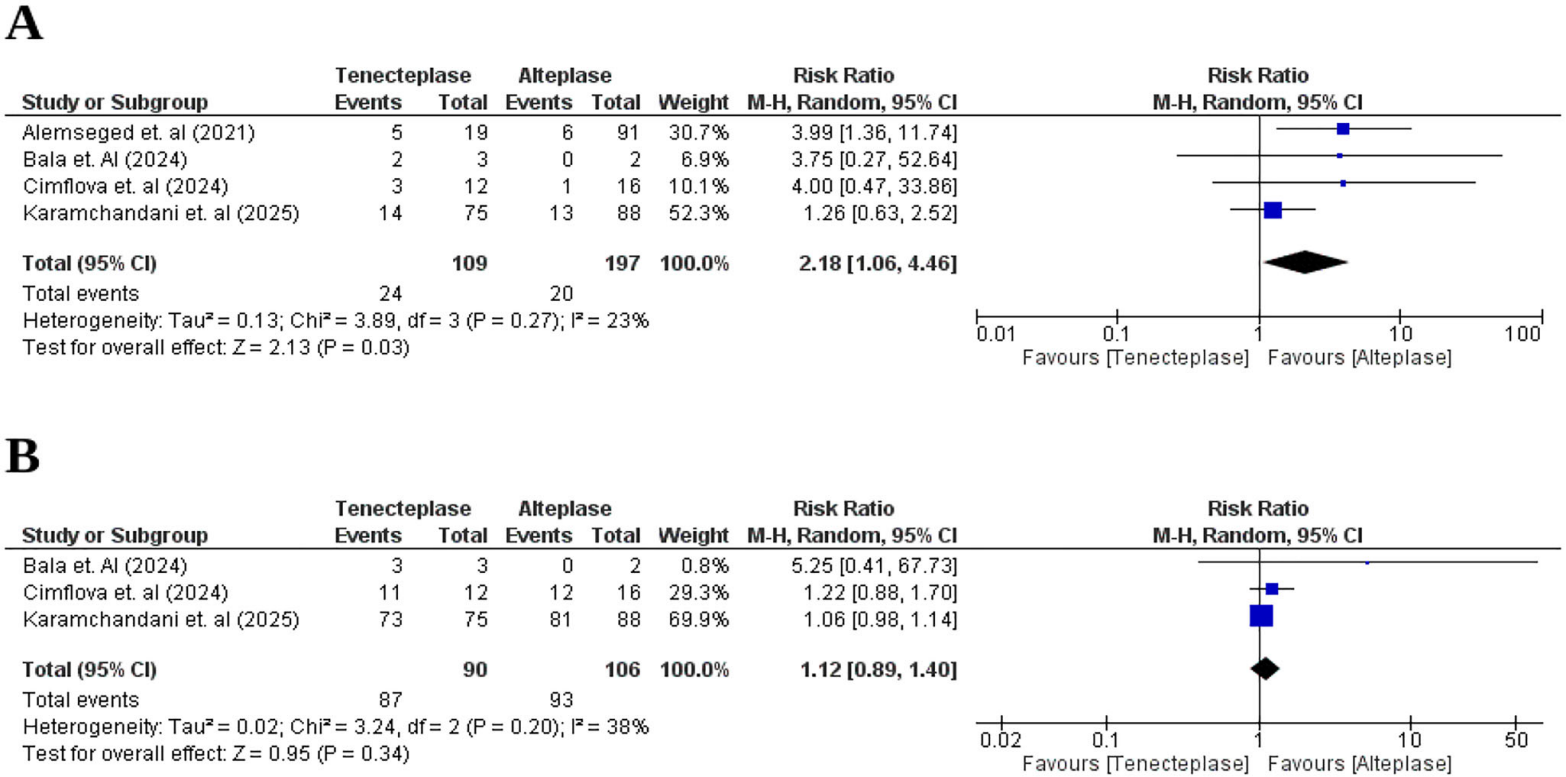
Forest plot comparing perfusion outcomes between tenecteplase versus alteplase.

#### 3.5.4 Post‐EVT Perfusion

Following endovascular therapy, post‐EVT perfusion success rates were comparable between the two treatments, failing to reach statistical significance (RR 1.12, 95% CI 0.89–1.40; *p =* 0.34; I^2^ = 38%) Figure 3.

### 3.6 Safety Outcomes

#### 3.6.1 Symptomatic Intracranial Hemorrhage (SICH)

Evaluating safety, the incidence of sICH was similar across groups and failed to reach statistical significance (RR 0.89, 95% CI 0.36–2.22; *p =* 0.81; I^2^ = 0%) Figure 4.

**FIGURE 4:**
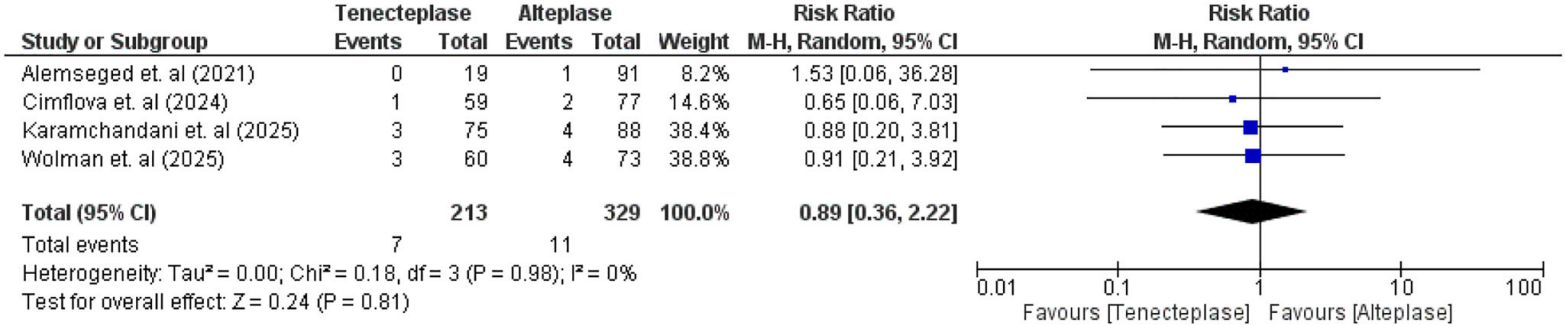
Forest plot comparing the incidence of symptomatic intracranial hemorrhage (sICH) for tenecteplase versus alteplase.

#### 3.6.2 Any Intracranial Hemorrhage

Rates of any intracranial hemorrhage were nearly identical between tenecteplase and alteplase, with no significant difference found (RR 1.02, 95% CI 0.59–1.78; *p =* 0.94; I^2^ = 0%) Figure 5.

**FIGURE 5:**
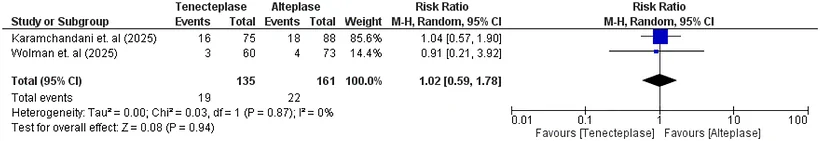
Forest plot comparing the incidence of any intracranial hemorrhage (ICH) between the two treatments.

#### 3.6.3 Any Parenchymal Hematoma

A slight reduction in parenchymal hematoma risk favored tenecteplase, although the results failed to reach statistical significance (RR 0.76, 95% CI 0.24–2.38; *p =* 0.64; I^2^ = 0%) Figure S1.

#### 3.6.4 90‐Day Mortality

Finally, 90‐day mortality rates favored tenecteplase slightly, but the findings remained statistically equivalent (RR 0.88, 95% CI 0.66–1.17; *p =* 0.39; I^2^ = 0%) Figure S2.

#### 3.6.5 Subgroup Analysis

Subgroup analyses demonstrated no significant differences between RCT/mixed PCIS and observational/BAO‐only studies for mRS 0–1, sICH, parenchymal hematoma, early reperfusion, final successful reperfusion, or 90‐day mortality (all *p* > 0.05). A significant subgroup difference was observed for mRS 0–2 (*p =* 0.02), with a significant benefit observed in observational/BAO‐only studies (RR 1.32, 95% CI 1.05–1.67; *p =* 0.02), but not in RCT/mixed PCIS studies (RR 0.90, 95% CI 0.71–1.14; *p =* 0.38) Figure S3–9.

### 3.7 Certainty of Evidence

The certainty of evidence for each outcome was evaluated using the GRADE approach. The certainty of evidence was rated as moderate for functional outcomes (mRS 0–3, mRS 0–2, mRS 0–1), pre‐ and post‐EVT perfusion, any intracranial hemorrhage, and 90‐day mortality, primarily due to concerns related to imprecision. In contrast, the certainty of evidence was judged to be low for sICH and any parenchymal hematoma due to very serious imprecision driven by wide confidence intervals and low event rates. Overall, the certainty of evidence was rated as low to moderate across the assessed outcomes. The detailed summary of findings is provided in Table S4.

## 4 Discussion

Ischemic stroke of the posterior circulation accounts for a substantial proportion of strokes and is associated with high morbidity and mortality due to brainstem involvement and large vessel occlusion. Most thrombolytic trials have focused primarily on anterior circulation strokes, leaving uncertainty about the optimal intravenous thrombolytic strategy for posterior circulation stroke. In this meta‐analysis, we specifically evaluated the comparative effectiveness of intravenous tenecteplase versus alteplase in patients with PCIS, thereby providing a focused synthesis of posterior circulation–specific functional, perfusion, and safety outcomes.

In this meta‐analysis, we pooled evidence from five studies, including randomized and observational/post hoc posterior circulation data. Overall, tenecteplase was associated with a 29% higher likelihood of achieving an ambulatory functional outcome at 90 days (mRS 0–3) compared with alteplase. However, this statistically significant finding was based on only two studies with a relatively small sample size, which limits the robustness and precision of the estimate and warrants cautious interpretation. For excellent (mRS 0–1) and good (mRS 0–2) functional outcomes, results favored tenecteplase; however, these differences did not reach statistical significance. Pre‐EVT perfusion was more than twice as likely in the tenecteplase group, which may suggest an association with greater early reperfusion. In contrast, post‐EVT perfusion outcomes were comparable between groups, suggesting no clear difference in final reperfusion success following intervention. Importantly, safety outcomes including sICH, any intracranial hemorrhage, parenchymal hematoma, and 90‐day mortality—were broadly similar between groups, with no meaningful increase in bleeding or mortality observed, although several estimates remain imprecise due to low event rates.

Compared with currently used thrombolytic strategies, tenecteplase has several practical and pharmacologic differences from alteplase in the treatment of acute ischemic stroke. Tenecteplase is a bioengineered variant of tissue plasminogen activator with amino acid substitutions that confer greater fibrin specificity, increased resistance to inhibition by plasminogen activator inhibitor‑1 (PAI‑1), and a significantly longer plasma half‑life than alteplase. These properties permit single‑bolus intravenous administration, rather than the bolus plus continuous infusion required for alteplase, and simplify preparation and dosing in emergency settings, potentially reducing workflow delays and medication errors (Miranda et al. 2025; Kobeissi et al. 2023). These mechanistic and workflow characteristics may partly contribute to the observed association with higher pre‐EVT perfusion and better ambulatory recovery in the present analysis. Many observational cohort studies have shown that tenecteplase is associated with significantly shorter door‑to‑needle and door‑to‑puncture times and a higher likelihood of meeting target acute stroke treatment benchmarks, as well as greater early neurological improvement at 24 hours, without increasing the risk of symptomatic or any intracranial hemorrhage compared with alteplase in routine clinical use (Yao et al. 2024; Henderson et al. 2025). A real‑world analysis by Maxhuni et al. reported similar early clinical outcomes and safety profiles (including sICH and functional status at discharge) between tenecteplase and alteplase during the initial implementation of tenecteplase in a stroke center, providing real‐world evidence of similar effectiveness and tolerability outside of controlled trials (Maxhuni et al. 2026). Moreover, large multicenter registry data from the CERTAIN collaboration by Warach et al. demonstrated that tenecteplase use in routine clinical practice was associated with significantly lower odds of sICH than alteplase, providing further evidence regarding its safety profile in unselected AIS patients (Warach et al. 2023).

Beyond the functional and safety outcomes highlighted earlier, additional clinical evidence has evaluated the potential role of tenecteplase as a viable alternative to alteplase in AIS. Several larger meta‑analyses and systematic reviews have examined aggregated data from randomized trials and observational studies. However, these broader AIS findings should be interpreted cautiously when applied to posterior circulation stroke, because vertebrobasilar occlusion has distinct pathophysiologic and prognostic features and remains underrepresented in most thrombolysis datasets. A recent meta‑analysis by Shen et al. which included over 7,508 patients across 16 randomized controlled trials, found that tenecteplase was not inferior to alteplase for early thrombolytic therapy and was associated with higher rates of early neurological improvement and vessel recanalization, without significant differences in safety outcomes such as sICH or mortality (Shen et al. 2023). Moreover, a systematic review pooling nine RCTs reported comparable rates of functional independence and 90‑day mortality between tenecteplase and alteplase, while indicating trends toward greater early neurological recovery with tenecteplase, although statistical significance varied across outcomes (Singh et al. 2024). Posterior circulation stroke has distinct anatomical and pathophysiological features that may contribute to differences in treatment response and clinical outcomes compared with anterior circulation stroke. Vertebrobasilar occlusions can involve the brainstem and cerebellum, where small lesions may produce substantial neurological deficits, while the extent of collateral circulation and variability in thrombus location and underlying atherosclerotic disease can influence tissue ischemia and reperfusion. In addition, the NIHSS may underestimate clinical severity in posterior circulation stroke because several relevant deficits are poorly captured by the scale (Salerno et al. 2021; Alemseged et al. 2021). These features may contribute to heterogeneity in functional and reperfusion outcomes; however, whether they specifically modify the relative effects of tenecteplase versus alteplase remains uncertain and requires further posterior circulation–specific investigation (Salerno et al. 2021; Alemseged et al. 2021; Ginsberg 2017). The higher pre‐EVT reperfusion observed with tenecteplase may reflect its greater fibrin specificity and longer half‐life, which could facilitate earlier thrombus dissolution before thrombectomy. Supporting this possibility, a retrospective analysis of patients with basilar artery occlusion reported pre‐EVT reperfusion in 26% of patients treated with tenecteplase compared with 7% with alteplase (RR 4.0, 95% CI 1.3–12; *p =* 0.02), although the evidence was observational and based on a small tenecteplase group (19 patients) (Alemseged and Campbell 2021). Once EVT is performed, final reperfusion is primarily determined by mechanical thrombectomy and may be influenced by procedural factors, thrombus characteristics, and vascular anatomy, potentially attenuating differences attributable to the preceding thrombolytic agent. Thus, the discrepancy between pre‐ and post‐EVT findings may indicate a potential association between tenecteplase and earlier pharmacologic reperfusion, while the effect on final reperfusion remains uncertain. The differential findings across mRS thresholds may reflect the broader definition of functional recovery represented by mRS 0–3. Whereas mRS 0–1 represents excellent functional recovery and mRS 0–2 represents functional independence, mRS 0–3 includes patients with moderate disability who remain able to walk without assistance. Thus, a treatment effect may be detectable at the broader ambulatory threshold without producing a sufficiently large difference in the proportion achieving excellent or independent recovery. This interpretation should, however, be considered cautiously because the mRS 0–3 analysis was based on only two relatively small studies. The limited number of studies and sample size also reduce statistical precision and raise the possibility that the observed significance may partly reflect random variation; therefore, the finding should be considered hypothesis‐generating and requires confirmation in larger, adequately powered studies (Weisscher et al. 2008).

Our findings are directionally consistent with these broader analyses but add more specific insight into the posterior circulation subgroup. Taken together, these findings suggest that tenecteplase may be associated with improved early reperfusion and ambulatory functional outcomes in PCIS; however, the limited number of studies, relatively small sample size for the mRS 0–3 analysis, and overall certainty of evidence preclude definitive conclusions regarding superiority over alteplase.

### 4.1 Limitations

This meta‐analysis has several limitations that should be acknowledged. First, only five studies with a total of 997 patients were included, which limits the overall statistical power and may reduce the generalizability of the findings. Second, the pooled evidence was derived from a mixture of randomized and observational data, which may introduce methodological heterogeneity despite acceptable quality assessment results. Third, some of the included studies were not originally designed exclusively for PCIS, and/or circulation data were extracted from subgroup analyses, which may weaken the strength of conclusions specific to this population. In addition, outcome definitions and reporting were not entirely uniform across studies, particularly for perfusion and recanalization‐related endpoints, which may have affected comparability between studies. Differences in treatment workflows, including onset‐to‐needle time, EVT use, and institutional stroke protocols, may also have influenced outcomes. Furthermore, several safety endpoints such as sICH and parenchymal hematoma were based on low event rates, resulting in wide confidence intervals and limiting precision. Additionally, the statistically significant finding for mRS 0–3 was derived from only two studies with a relatively small sample size, limiting the robustness of this estimate and warranting cautious interpretation. Finally, the analysis relied on study‐level rather than individual patient‐level data, which restricted more detailed subgroup analyses according to stroke severity, occlusion site, thrombolytic dose, and timing of reperfusion therapy.

## 5 Conclusion

In patients with PCIS, intravenous tenecteplase was associated with improved ambulatory functional outcome (mRS 0–3) and higher pre‐EVT perfusion compared with alteplase, while other functional, perfusion, and safety outcomes were broadly similar between treatments. These findings suggest that tenecteplase may be an effective and safe alternative to alteplase in this subgroup. However, the evidence remains limited by small sample size, inclusion of mixed study designs, and low event rates for several safety endpoints. Larger posterior circulation–specific prospective studies are needed to confirm whether the apparent early reperfusion and ambulatory outcome advantages of tenecteplase translate into consistent long‐term clinical benefit.

## Author Contributions

**Shaheer Bin Shafiq**: conception and design of the study. **Ammad Uddin**: conception and design of the study. **Muhammad Hussain**: analysis and interpretation of data. **Ghulam Taha Khan**: collection or assembly of data. **Muhammad Hassan**: analysis and interpretation of data. **Muhammad Junaid Razzak**: analysis and interpretation of data. **Mominah Majid**: conception and design of the study. **Faisal Islam**: drafting or revising the article. **Umer Ahmed**: final approval of the version to be published. **Ahmed Anwaar Uddin**: drafting or revising the article. **Hasibullah Aminpoor**: final approval of the version to be published. **Muhammad Ahmed**: final approval of the version to be published.

## Funding

The authors have nothing to report.

## Conflicts of Interest

The authors declare no conflicts of interest.

## Supporting information




**Supplementary Material**: brb371831‐sup‐0001‐SuppMat.docx

## Data Availability

The data supporting the findings of this study were obtained from published studies. The data and material used were publicly available.
