# Supplementary material for: Comparative Effectiveness of Intravenous Tenecteplase versus Alteplase in Posterior Circulation Ischemic Stroke: A Systematic Review and Meta‐Analysis
Source: Brain Behav. 2026 Oct 8;16(10):e71831. doi: 10.1002/brb3.71831 (PMC13650503; doi:10.1002/brb3.71831)
Supplement: Supplementary file 1 [file BRB3-16-e71831-s001.docx]

**SUPPLEMENTARY FILE**

Supplementary Table 1**.** Search strategy used for obtaining studies

(Tenecteplase OR Metalyse OR TNKase OR "TNK tPA" OR "TNK-tPA" OR Elaxim)

AND

(Alteplase OR Actilyse OR Activase OR Cathflo OR "Tissue plasminogen activator" OR rtPA OR tPA)

AND

("Ischemic stroke" OR "Ischaemic Stroke" OR "Acute Ischemic Stroke" OR "Cerebral Infarction" OR Stroke OR Thrombectomy OR "Posterior circulation" OR vertebrobasilar OR "basilar artery" OR BAO)

Supplementary Table S2. Risk of Bias Summary.

*Risk of bias assessment was performed for the parent randomized controlled trials (RCTs). Campbell et al. 2018 represents the EXTEND-IA TNK trial, Campbell et al. 2020 represents EXTEND-IA TNK Part 2, and Menon et al. 2022 represents the AcT trial. The included studies by Cimflova et al. (2024), and Bala et al. (2024) were secondary/post-hoc analyses derived from these parent RCTs; therefore, RoB 2 assessments reflect the original trials rather than the post-hoc analyses.*

Supplementary Table 3**.** Risk of Bias for observational studies NOS.

| **Studies** | **Selection** | | | | **Comparability** | | **Exposure** | | | **Total Quality score** |
| --- | --- | --- | --- | --- | --- | --- | --- | --- | --- | --- |
|  | Representativeness of exposed cohort | Selection of non-exposed cohort | Ascertainment of exposure | Outcome not present at start | Control for most important confounders | Control for additional confounders | Assessment of outcome | Follow-up long enough for outcomes | Adequacy of follow-up |  |
| Wolman et al 2025 | * | * | * | * | * | - | * | * | - | 7 |
| Karamchandani et. al (2025) | * | * | * | * | * | - | * | * | * | 8 |
| Alemseged et al (2021) | * | * | * | * | * | * | * | * | * | 9 |

Supplementary Table 4: GRADE summary of findings table for efficacy and safety outcomes

| Certainty assessment | | | | | | | Summary of findings | | | | |
| --- | --- | --- | --- | --- | --- | --- | --- | --- | --- | --- | --- |
| Participants (studies) Follow-up | Risk of bias | Inconsistency | Indirectness | Imprecision | Publication bias* | Overall certainty of evidence | Study event rates (%) | | Relative effect (95% CI) | Anticipated absolute effects | |
|  |  |  |  |  |  |  | With Alteplase | With Tenecteplase |  | Risk with Alteplase | Risk difference with Tenecteplase |
| mRS 0–1 at 90 Days | | | | | | | | | | | |
| 433 (4 studies) | not serious | not serious | not serious | serious | undetected | ⨁⨁⨁◯ Moderate | 90/272 (33.1%) | 60/161 (37.3%) | RR 1.19 (0.91 to 1.56) | 90/272 (33.1%) | 63 more per 1,000 (from 30 fewer to 185 more) |
| mRS 0–2 at 90 Days | | | | | | | | | | | |
| 587 (5 studies) | not serious | not serious | not serious | serious | undetected | ⨁⨁⨁◯ Moderate | 170/358 (47.5%) | 123/229 (53.7%) | RR 1.10 (0.89 to 1.37) | 170/358 (47.5%) | 47 more per 1,000 (from 52 fewer to 176 more) |
| mRS 0–3 at 90 Days (Ambulatory) | | | | | | | | | | | |
| 279 (2 studies) | not serious | not serious | not serious | serious | undetected | ⨁⨁⨁◯ Moderate | 74/157 (47.1%) | 74/122 (60.7%) | RR 1.29 (1.03 to 1.60) | 74/157 (47.1%) | 137 more per 1,000 (from 14 more to 283 more) |
| Mortality at 90 Days (mRS 6) | | | | | | | | | | | |
| 477 (4 studies) | not serious | not serious | not serious | serious | undetected | ⨁⨁⨁◯ Moderate | 79/267 (29.6%) | 55/210 (26.2%) | RR 0.88 (0.66 to 1.17) | 79/267 (29.6%) | 36 fewer per 1,000 (from 101 fewer to 50 more) |
| Symptomatic ICH (sICH) | | | | | | | | | | | |
| 542 (4 studies) | not serious | not serious | not serious | very serious | undetected | ⨁⨁◯◯ Low | 11/329 (3.3%) | 7/213 (3.3%) | RR 0.89 (0.36 to 2.22) | 11/329 (3.3%) | 4 fewer per 1,000 (from 21 fewer to 41 more) |
| Any Intracranial Hemorrhage | | | | | | | | | | | |
| 296 (2 studies) | not serious | not serious | not serious | serious | undetected | ⨁⨁⨁◯ Moderate | 22/161 (13.7%) | 19/135 (14.1%) | RR 1.02 (0.59 to 1.78) | 22/161 (13.7%) | 3 more per 1,000 (from 56 fewer to 107 more) |
| Any Parenchymal Hematoma (PH) | | | | | | | | | | | |
| 335 (3 studies) | not serious | not serious | not serious | very serious | undetected | ⨁⨁◯◯ Low | 9/212 (4.2%) | 4/123 (3.3%) | RR 0.76 (0.24 to 2.38) | 9/212 (4.2%) | 10 fewer per 1,000 (from 32 fewer to 59 more) |
| Early Reperfusion | | | | | | | | | | | |
| 306 (4 studies) | not serious | not serious | not serious | serious | undetected | ⨁⨁⨁◯ Moderate | 20/197 (10.2%) | 24/109 (22.0%) | RR 2.18 (1.06 to 4.46) | 20/197 (10.2%) | 120 more per 1,000 (from 6 more to 351 more) |
| Final Successful Reperfusion | | | | | | | | | | | |
| 196 (3 studies) | not serious | not serious | not serious | serious | undetected | ⨁⨁⨁◯ Moderate | 93/106 (87.7%) | 87/90 (96.7%) | RR 1.12 (0.89 to 1.40) | 93/106 (87.7%) | 105 more per 1,000 (from 97 fewer to 351 more) |

**Publication bias could not be formally assessed for any outcome because fewer than ten studies contributed to each analysi*

Supplementary Figure 1. Forest plot assessing the occurrence of parenchymal hematoma for Tenecteplase and Alteplase.

Supplementary Figure 2. Forest plot comparing the 90-day mortality rates between Tenecteplase and Alteplase.

# Supplementary Figures: Subgroup Analyses

Supplementary forest plots presenting subgroup analyses according to study design and stroke population. These analyses compare RCT/mixed-PCIS studies with observational/BAO-only studies as described in the manuscript.

Supplementary Figure 3. Subgroup analysis of excellent functional outcome (mRS 0–1) at 90 days.

Supplementary Figure 4. Subgroup analysis of functional independence (mRS 0–2) at 90 days.

Supplementary Figure 5. Subgroup analysis of symptomatic intracranial hemorrhage (sICH).

Supplementary Figure 6. Subgroup analysis of parenchymal hematoma (PH).

Supplementary Figure 7. Subgroup analysis of early reperfusion.

Supplementary Figure 8. Subgroup analysis of final successful reperfusion.

Supplementary Figure 9. Subgroup analysis of 90-day mortality.
